# Coarse-Grained Modeling of On-Surface Self-Assembly of Mixtures Comprising Di-Substituted Polyphenyl-Like Compounds and Metal Atoms of Different Sizes

**DOI:** 10.1021/acsomega.1c02857

**Published:** 2021-09-21

**Authors:** Łukasz Baran

**Affiliations:** Department of Theoretical Chemistry, Maria Curie Skłodowska University, 20-031 Lublin, Poland

## Abstract

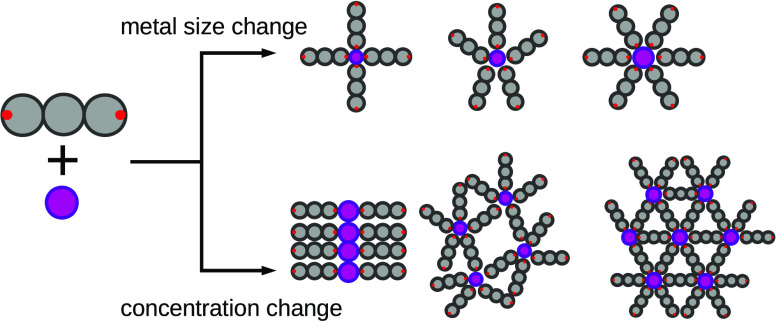

We use coarse-grained molecular dynamics simulations to investigate the phase behavior of binary mixtures of di-substituted polyphenyl-like compounds and metal atoms of different sizes. We have estimated the possible on-surface behavior that could be useful for the target design of particular ordered networks. We have found that due to the variation of system conditions, we can observe the formation of the parallel, square, and triangular networks, Archimedean tessellation, and “spaghetti wires.” All of these structures have been characterized by various order parameters.

## 1 Introduction

Fabrication of two-dimensional materials attracts considerable attention, owing to their possibility to exhibit different features from their bulk counterparts. This field has begun with the discovery of graphene and characterization of its properties, especially in the electronic field.^1^ From this date, a variety of different two-dimensional (2D) materials have been synthesized, and two main routes have been established. The first one is a top-down approach that benefits from the general knowledge of the three-dimensional (3D) materials such as covalent or metal-organic frameworks (COFs and MOFs, respectively) and is supposed to exfoliate a layered crystal due to applied external forces to form a single layer of the smallest thickness as possible. The second protocol is a bottom-up approach, which can be applied on the surfaces such as highly oriented pyrolytic graphite (HOPG) or coinage metals (Au, Ag, Cu) or in the air/water or liquid/liquid interfaces. The obtained single nanolayers have already been used as membranes for separation in both liquid and gas phases,^2^ batteries,^3^ molecular sieves,^4^ and insulin delivery.^5^

The on-surface synthesis performed either in ultrahigh vacuum or liquid conditions generally has proven to be the successful and most conventional routine for the preparation of well-ordered networks. To date, a variety of compounds of different geometry have been investigated, and it has been found that they can form small clusters^6^ up to extended porous structures,^7^ as well as Kagomé patterns,^8,9^ rhombus tilings,^10^ and five-vertex Archimedean tessellations.^11,12^ The latter is particularly interesting since it has been obtained in a mixture of dicarbonitrile polyphenyl compounds with rare-earth metal atoms, whereas the first reports of such structures appeared in alloy particles^13^ or chalcogenides.^14^

In this paper, we wanted to further explore the conditions on how di-substituted polyphenyl-like (linker) molecules behave with mixtures of metal atoms. Unlike in the references,^11,12^ we have changed not only the mixture concentration but also the metal atom sizes. For this purpose, we have designed a coarse-grained model and performed comprehensive molecular dynamics simulations. We believe that the protocol used in the course of this study can give a very helpful insight for the experimentalists owing to the fact that computer modeling is a very convenient substitute to the exploration of problems of interest and can reasonably complement experimental findings. Although there are other methods that have been widely used such as quantum density functional theory^15^ or classical Monte Carlo,^16−21^ we have already proven that the approach used in our laboratory can be useful for examination of similar systems of interest both in one-component systems and binary mixtures.^22−24^

## 2 Methods

**Figure 1:**
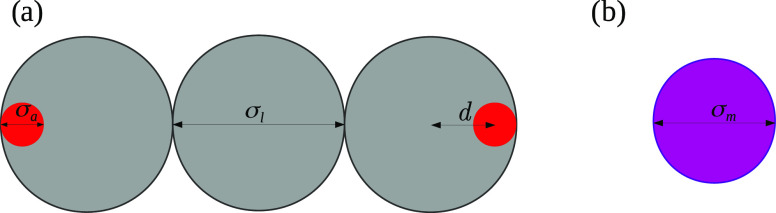
Model of the linker molecule (a) and metal atom (b). Gray circles correspond to the segments of the linear linker, whereas the red ones pertain to the active sites. For the description of parameters used in the model, confer the text.

In this paper, the geometry of the linear linker molecules has been devoted to reflecting the behavior of di-substituted polyphenyl compounds, as shown in Figure 1. In its structure, each of the gray segments mimicked one phenyl group, whereas red entities were the active interaction centers. The size of every linker’s segment has been set to σ_*l*_ = σ, while the active sites have been five times smaller, σ*_a_* = 0.2σ_*l*_. The segments in the former have been tangentially jointed with one another; therefore, the bonding distance has been set to σ_*l*_. The active sites have been entirely embedded into both terminal units of the linear linker, and the bonding distance has been abbreviated as *d* = 0.36σ_*l*_. In our previous paper, we have already shown that both the size and the bonding distance *d* can provide structures of 3- to 6-fold symmetries.^22^ This approach, however, lacks the possibility to change the concentration of the mixtures since the second component has been treated implicitly. Therefore, we wanted to fill this gap, and metal atoms in our simulations have been treated explicitly, and their size varied between σ_*m*_ = 0.5 – 1.0σ_*l*_.

In molecular dynamics simulations, all of the objects have been treated as flat and rigid objects, and all of the necessary bonds have been maintained by harmonic binding potentials
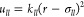
1and
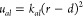
2Likewise, all of the necessary angles have been preserved
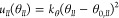
3and
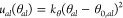
4The interparticle potential employed in our simulations was (12,6) Lennard-Jones potential, which has been appropriately shifted to ensure the continuity of both the potential and of its first derivative^25,26^
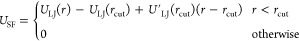
5where *U*_LJ_(*r*) = 4ε_*ij*_[(σ_*ij*_/*r*)^12^ – (σ_*ij*_/*r*)^6^] and *U*′_LJ_(*r*_cut_) is the first derivative of *U*_LJ_(*r*) at *r* = *r*_cut_.

The Lennard-Jones potential parameters, σ_*l*_ = σ and ε_*ll*_ = ε, have been set to be the units of length and energy, respectively. The reduced time and temperature are equal to  and T* = *kT*/ε_*ll*_, respectively. The number density has been defined as , where *R* and *N*_*m*_ are the number of segments in the linear linker and metal “atoms,” respectively. Moreover, we define the binary mixture composition as , where *N*_*l*_ is the number of linker molecules and *N*_tot_*= N*_*l*_ + *N*_*m*_. It has been varied in the range of χ *=* 0.25 – 0.83.

The energies of the linker–linker and the linker–active site interactions have been set to ε_*ll*_ = ε_*aa*_ = ε and ε_*ma*_ = 5.0ε. The linker-site diameter and the energy of the linker-site interactions have been set to σ_*al*_ = (σ_*a*_ + σ_*l*_) /2 and ε_*al*_ = ε, respectively. The cutoff distance of the interactions between the active site and the metal atom has been set to *r*_cut,*ma*_ = 2σ*_ma_*, whereas the remaining ones are *r*_cut,*ij*_ = σ_*ij*_, where *ij* = *aa*, *al*, *ll*, *lm*, and *mm*. This has been done to assume that the only attraction in the system is due to the metal-organic coordination, whereas the remaining are the soft-core interactions. We did not use any solvent explicitly, but rather by means of presented interparticle potential, we modeled the system so that the interactions other than the active site-metal atoms are screened due to the solvent presence. The harmonic potential constants *k*_*al*_ ≡ *k*_*ll*_ have been set to 1000ε/σ^2^ and *k*_θ_ = 1000ε/(rad)^2^. Such high values of harmonic constants have been set to reduce the range of fluctuations and, in consequence, to maintain the rigidity of the assumed geometries.

All of the molecular dynamics simulations have been performed in the NVT ensemble, using LAMMPS simulation package.^27,28^ The velocity Verlet integration scheme has been used with the reduced time step of the order of *t* = 0.001τ. The number of linker molecules and metal atoms varied from 1600 to 8000 and 1600 to 4800, respectively. However, one has to note that the total number of atoms varied, depending on the concentration. This amount is sufficient for most of the self-assembly systems, which is simultaneously large enough to form ordered networks and small enough to form those structures in a reasonable time frame.

The simulation scheme involved preliminary runs in the NPT ensemble to establish the desired density. Next, equilibration runs for 5 × 10^6^ times steps using Berendsen thermostat,^29^ with the damping constant equal to τ_B_ = 10τ have been performed. Further equilibration for 5 × 10^7^ as well as production runs have been performed using Nosé–Hoover chain algorithm,^30^ with the damping constant equal to τ_NH_ = 10τ and the number of chains set to *N*_chain_ = 3. Every system has been cooled down from temperatures where we did not observe any order, up to the point where self-assembled networks have been distinct. The temperature grid was set to Δ T* = 0.01.

## 3 Results and Discussion

**Figure 2:**
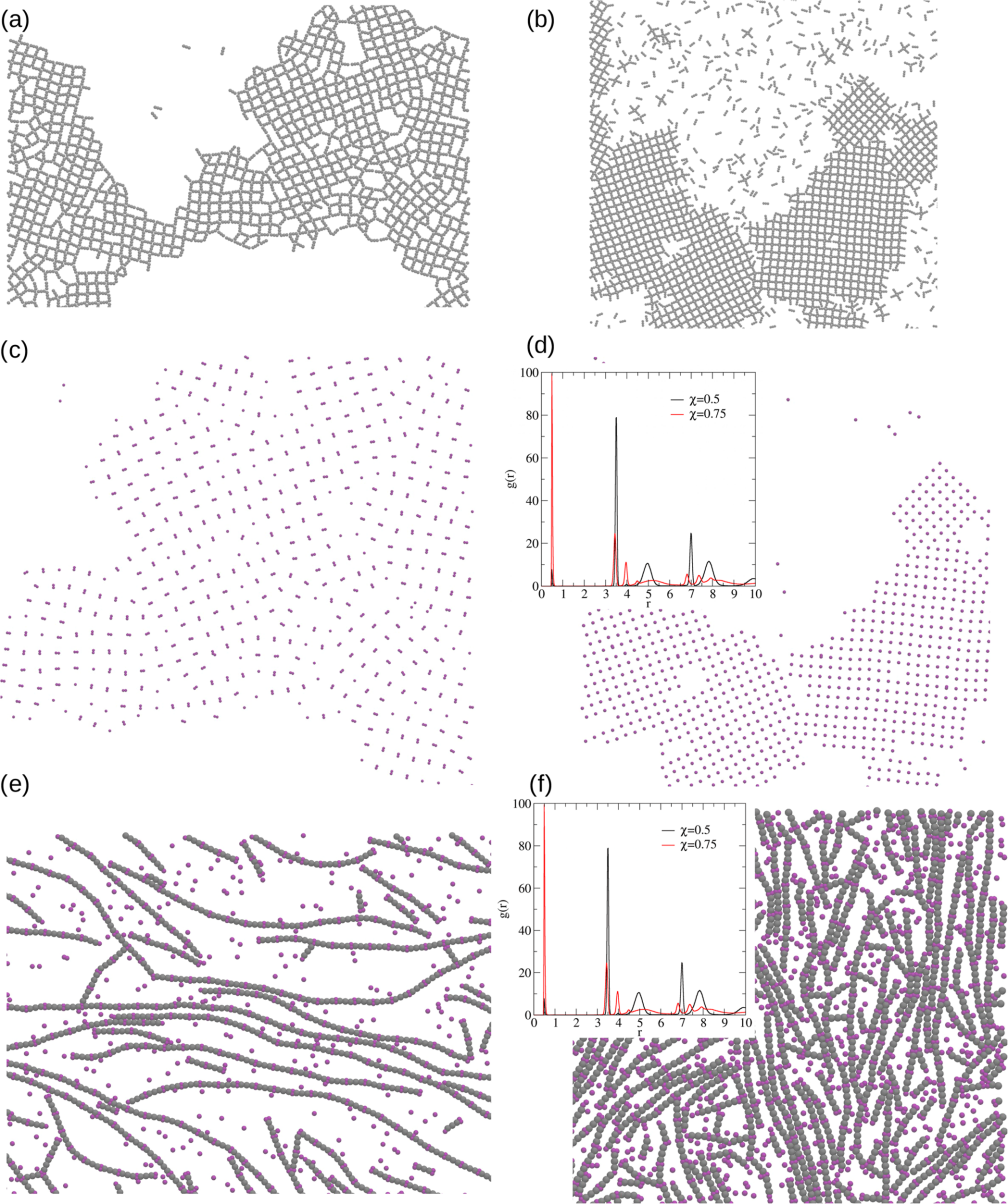
Fragment of the configurations for linker molecules (a, b) and metal atoms of size σ_*m*_ = 0.5σ (c, d) for χ = 0.5 (a, b) and χ = 0.75 (c, d) mixture compositions in ρ* = 0.2 at T* = 0.3. Fragment of the configurations for χ = 0.25 in ρ* = 0.2 at T* = 0.3 (e) and in ρ* = 0.5 at T* = 0.4 (f). The insets to part (d) and (f) display the radial distribution function calculated with respect to metal atoms.

Let us start from the description of the binary mixture with metal atoms 2-fold smaller than the diameter of core’s segments, i.e., σ_*m*_ = 0.5σ. The results for the system with an equal amount of linker and metal entities (χ = 0.5) can be found in Figure 2a. One can see the formation of a network with square symmetry with distinct imperfections in its structure. If one increases the number of linker molecules three times (χ = 0.75), the formation of a nearly perfect square lattice can be observed, as it has been shown in Figure 2b. To better understand the development of this network, we wanted to investigate the arrangement of metal atoms. In part (c) of Figure 2, we can see their layout for the mixture composition χ = 0.5. In this case, two atoms tend to glue with one another, despite their soft-core interactions. On the contrary, for χ = 0.75, metal atoms are entirely separated (cf. Figure 2d).

To verify if observations from snapshots are correct, we have calculated the radial distribution function with respect to metal atoms, which can be found in the inset to Figure 2d. For the smaller molar fraction χ = 0.5, the most prominent peak is around *r* ≈ 0.5, which means that those entities are glued one to another. On the other hand, for higher χ = 0.75, this peak almost vanished, and the most prominent distance is around *r* ≈ 3.5. Moreover, we have computed the number of dimers in both cases, which is approximately 90 and 5% for mixture compositions χ = 0.5 and 0.75, respectively.

Another quantity that we used to characterize the formation of a highly ordered, square network was the two-dimensional bond-orientational order parameter (BOOP), calculated with respect to metal atoms, which is defined as^31^
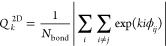
6where *i* runs over all metal atoms of the system, *j* runs over all neighbors of *i*, ϕ_*ij*_ denotes the angle between the bond connecting particles *i* and *j* and an arbitrary but fixed reference axis, *N*_bond_ denotes the number of bonds in the system, and *k* = 2, 3, 4, 5, 6. For the square network, we have assumed that two metal atoms are neighbors if their distance is less than 3.8σ, which is the second minimum extracted from the radial distribution function (cf. inset to Figure 2d). The bond-orientational order parameter can take the values between 0 and 1 for the disordered and the ordered structures of a particular symmetry, respectively.

To corroborate the observations from snapshots and radial distribution function, we have calculated this parameter for the aforementioned mixture compositions. In the first case, i.e., χ = 0.5, the 2D BOOP is approximately *Q*_4_ = 0.202 ± 0.05, which indicates that there is an order to some extent, however, the presence of imperfections in the network is noticeable, which in consequence decreases its value. On the other hand, for the composition χ = 0.75, this parameter takes a value of *Q*_4_ = 0.915 ± 0.03, which corresponds to a nearly perfect structure of 4-fold symmetry. This analysis demonstrates that the increase in the number of linker molecules in the system stabilizes the formation of a square network.

We have also examined the mixture composition of χ = 0.25, which means that there are 3-fold more metal atoms than linker molecules. In this case, we have found that the formation of “spaghetti-like” strings (cf. Figure 2e). Similarly, as in the case of χ = 0.5, metal atoms are gluing one to another and are forming “dimers”. An increase of the density does not lead to the increase of order in the system, and those strings do not start to align in one direction (cf. Figure 2f). The radial distribution function calculated with respect to metal atoms shown in the inset to Figure 2f shows that the most prominent peak is around *r* ≈ 0.5, which confirms that metals tend to form dimers. Moreover, we have computed the number of dimers in both cases, which is approximately 63 and 53% for the densities ρ* = 0.2 and 0.5, respectively.

**Figure 3:**
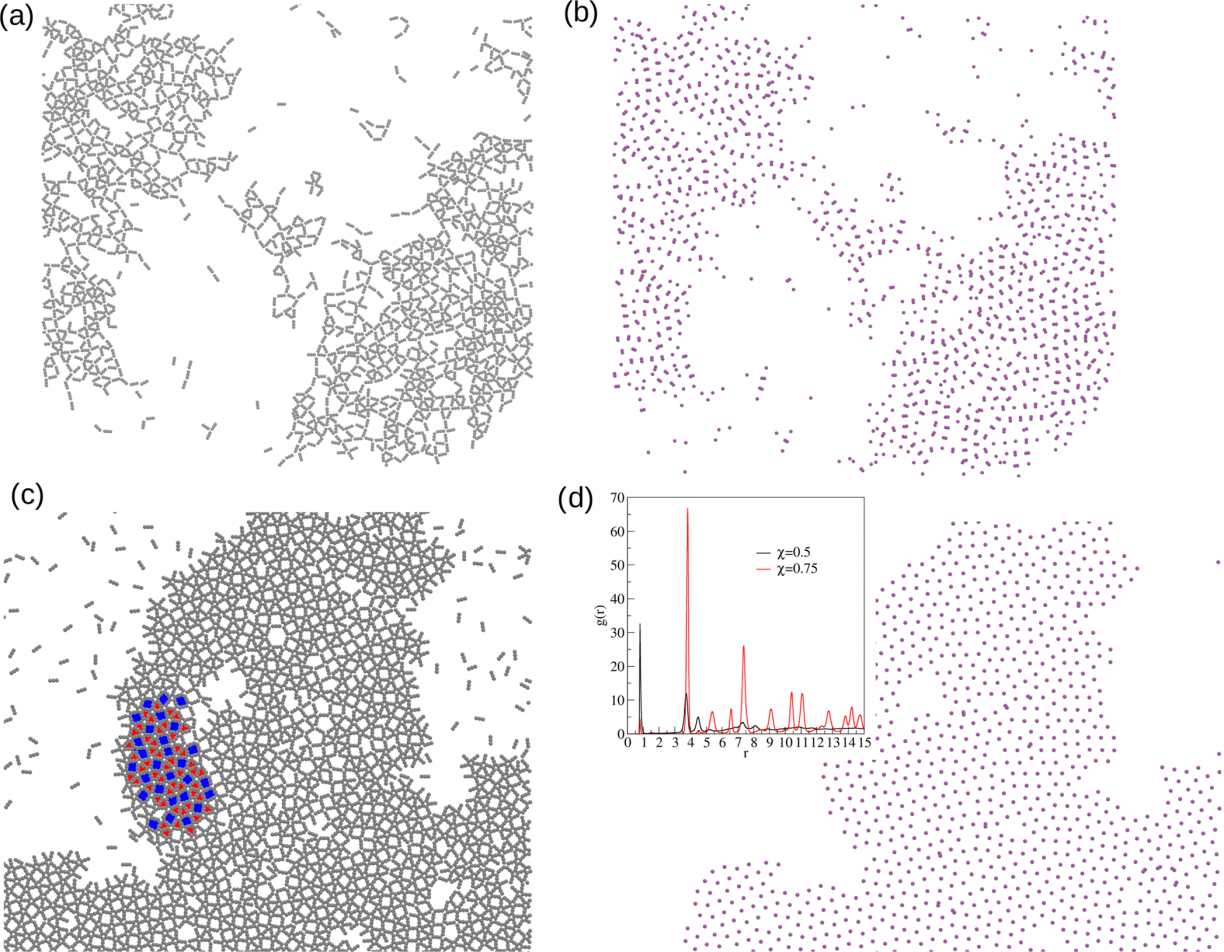
Fragment of the configurations for linker molecules (a, c) and metal atoms of size σ_*m*_ = 0.8σ (b, d) for mixture compositions χ = 0.5 in ρ* = 0.2 at T* = 0.4 (a, c) and χ = 0.75 in ρ* = 0.2 at T* = 0.3 (b, d). The inset to part (d) displays the radial distribution function calculated with respect to metal atoms.

Next, we proceed to the description of a binary mixture with metal atoms of size equal to σ_*m*_ = 0.8σ. The results for χ = 0.5 can be found in Figure 3a. One can see that we are not able to distinguish any network of a particular symmetry. Linker molecules connect with metal atoms quite randomly, and multiple pore shapes can be observed. The arrangement of metal atoms as shown in Figure 3b shows that for this mixture composition, they tend to glue one to another and form dimers, as for smaller metal sizes. As previously, it leads to the disturbance in the formation of any ordered network.

The results for the system with mixture composition χ = 0.75 can be found in Figure 3c. In this case also, the formation of multiple pore shapes can be observed; however, this pattern resembles the 3^2^.4.3.4 Archimedean tiling with several visible imperfections. For better visualization, we have colored the particular polygons belonging to this semiregular tessellation. The arrangement of metal atoms, as shown in Figure 3d, shows that they are separated, as it has been observed in a previous case (cf. Figure 2). The radial distribution function inserted to part (d) of this figure corroborates with the observations from the snapshots. Likewise, as for smaller σ_*m*_, we have evaluated the average amount of dimers in the system, which is approximately 73% (χ = 0.5) and 4% (χ = 0.75). We conclude that the increase of the number of linker molecules leads to the stabilization of ordered networks of a particular symmetry.

Similarly, as for the previous metal size, we have examined the mixture composition of χ = 0.25. The formation of similar spaghetti stripes has been found, as in the case of σ_*m*_ = 0.5σ. The results have been omitted for the sake of brevity.

**Figure 4:**
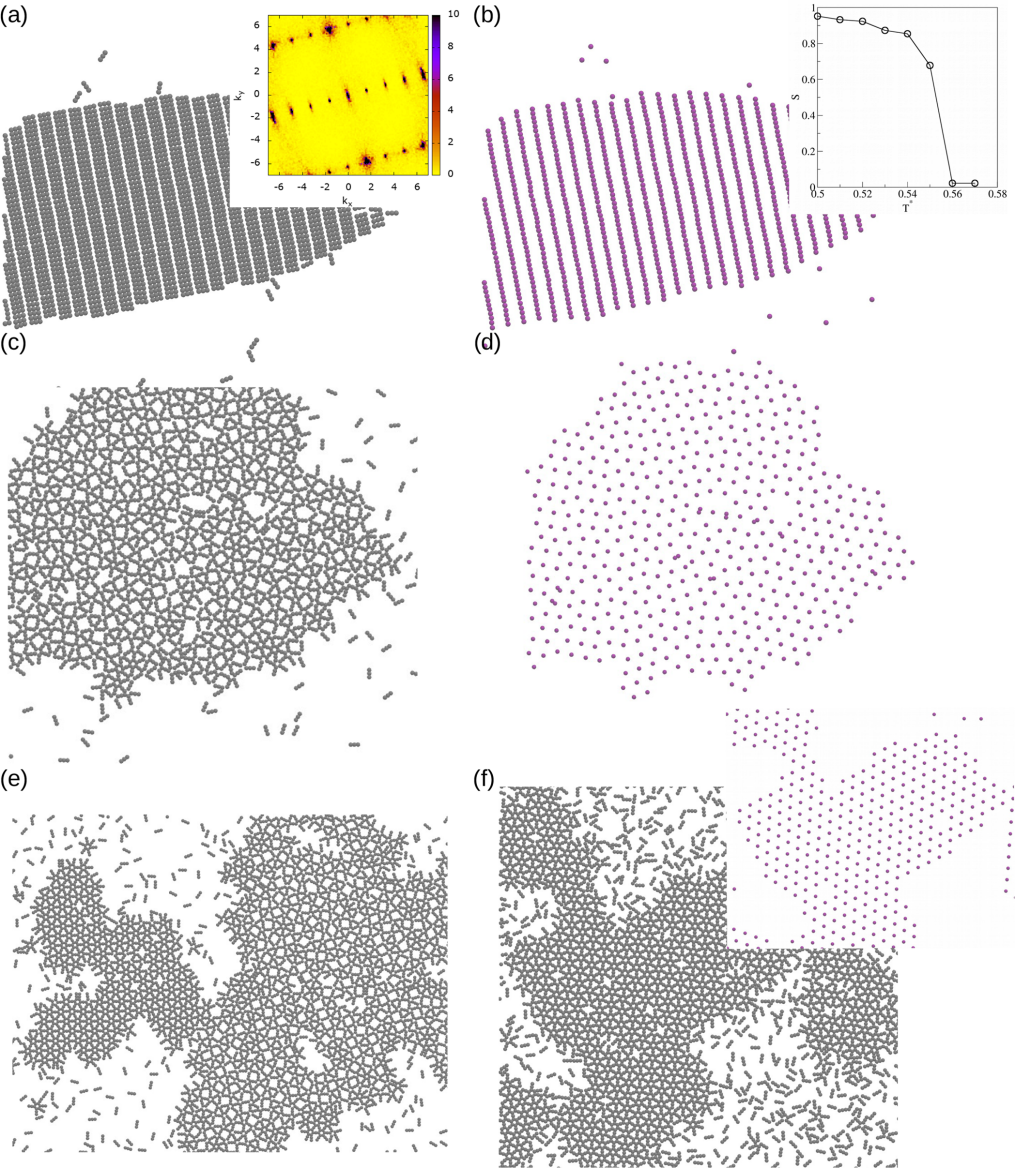
Fragment of the configurations for linker molecules (a, c) and metal atoms of size σ_*m*_ = 1.0σ (b, d) for mixture compositions χ = 0.5 in ρ* = 0.2 at T* = 0.5 (a, b) and χ = 0.75 in ρ* = 0.2 at T* = 0.4 (c, d). The insets to part (a) and (b) show the 2D structure factor and relation of nematic order parameter with respect to the temperature, respectively. Parts (e) and (f) display the snapshots for the systems χ = 0.83 in ρ* = 0.2 at T* = 0.3 and χ = 0.83 in ρ* = 0.4 at T* = 0.4, respectively. The inset to part (f) displays the arrangement of metal atoms in this system.

Let us proceed to the description of a binary mixture with metal atoms of size equal to σ_*m*_ = 1.0σ. The results for the system with mixture composition χ = 0.5 can be found in Figure 4a. In this case, we can see the formation of a network with both positional and orientational order. To prove the former, we have calculated the two-dimensional structure factor^24^ with respect to linker molecules, which can be found in the inset to Figure 4a. Moreover, to demonstrate the orientational order, we have calculated the nematic order parameter^32^ with respect to linker molecules, defined as
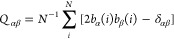
7where *b*_α_(*i*) is the α-th coordinate of the unit vector *b*, specifying the orientation of the molecule *i*, and δ_αβ_ is the Kronecker delta function. The corresponding eigenvalues of *Q* are ±*S*. This function takes values between 0 and 1 in disordered and perfectly ordered phases, respectively. In real systems, it is very difficult to reach the value of *S* equal to 1, owing to the possible imperfections of the ordered structure or rotation of differently oriented domains.

One can see that for this structure, the value of this order parameter is around *S* ≈ 0.95 in the lowest temperatures. This value proves the formation of a highly ordered network of a single orientation. The temperature relation of this quantity also indicates that the structure remains until T* = 0.56.

The results for the system with mixture composition χ = 0.75 can be found in Figure 4c. We can see the formation of similar 3^2^.4.3.4 Archimedean tiling, as for smaller metal size. It is noteworthy that this structure has more visible imperfections compared to the previous case.

Further increase of linker molecules in relation to metal atoms, i.e., χ = 0.83, not only leads to the formation of 3^2^.4.3.4 Archimedean tiling but also a network with triangular symmetry can be observed. An increase in the density of the system shows that the semiregular tessellation vanished, and the latter structure is only present. The BOOP for this network takes high values and is around *Q*_6_ = 0.96. This indicates that the Archimedean tessellation in this system is not a stable structure, and the formation of a triangular network is favored. However, it is worth mentioning that the same situation can be observed in experiments, where the formation of various different patterns can be observed, and the determination of which of them is thermodynamically stable is not so trivial.

Finally, we proceed to the examination of the system with the mixture composition of χ = 0.25. Surprisingly, we do not observe the formation of spaghetti wires, as for previous cases, but the parallel network remains. The only effect that the increase of the number of metal atoms caused is that there are two differently ordered domains in the system. In this case, due to the observation of two differently oriented domains, the nematic order parameter takes values around *S* ≈ 0.55. However, it is worth mentioning that if one would compute this quantity separately for each of those clusters, the situation would reflect the one observed in Figure 4a. The corresponding snapshots have been omitted for the sake of brevity.

## 4 Conclusions

**Figure 5:**
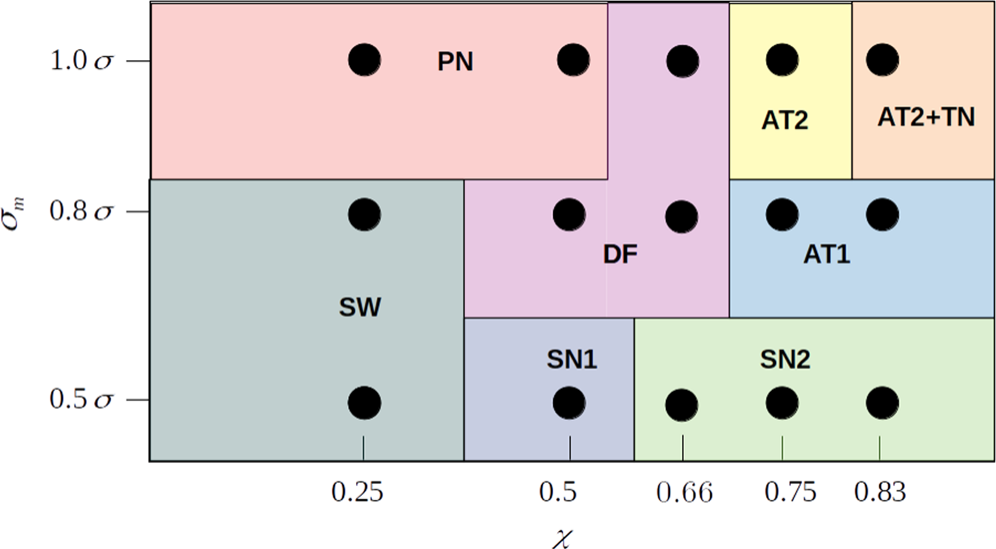
Schematic overview of structures formed in the binary mixtures investigated in this study. Black circles refer to simulation results; structure boundaries are drawn arbitrarily to guide the eye.

In this paper, we have investigated the phase behavior of binary mixtures of di-substituted polyphenyl-like molecules and metal atoms. We considered the influence of metal atoms’ size and the mixture composition of the self-assembly behavior. To deepen our discussion, we summarize the results in a more systematic way. In Figure 5, we present the overview of the structures observed for the systems with different metal atom sizes σ_*m*_ and mixture compositions χ.

We have found that for σ_*m*_ = 0.5σ, depending on the mixture composition χ, the formation of two distinct networks can occur, which are spaghetti wires (SW) (cf. Figure 2e,f) and a nearly perfect square network (SN2) (cf. Figure 2b). The imperfect square structure (SN1) (cf. Figure 2a) is quite similar to SN2, but due to the concentration χ, the metal atoms form dimers, which in consequence, result in the deterioration of the formed square lattice. We conclude that the mixture composition below a certain amount of linker molecules enforces gluing metal atoms with one another, which may lead to a bigger amount of possible orientations on how linker molecules can interact with them. This corroborates with the observation that a nearly perfect square network SN1 is formed in higher linker concentrations due to the separation of metal atoms.

An increase of metal size to σ_*m*_ = 0.8σ leads to the formation of similar spaghetti wires as for σ = 0.5σ; however, the ordered network is completely different. We have observed the development of 3^2^.4.3.4 Archimedean tessellation (AT1) for the mixture concentrations of χ = 0.75 and above (cf. Figure 3c). Similarly, as for the previous case, the formation of the ordered network was only possible if the mixture composition enforced the separation of metal atoms.

For the further increase to σ_*m*_ = 1.0σ, we observe the occurrence of a parallel network (PN) for mixture composition of χ = 0.25 and 0.5 (cf. Figure 4a). In higher concentrations of linker molecules, we can see two different types of ordered structures. The first one is similar to 3^2^.4.3.4 Archimedean tessellation; however, we observe significant imperfections in its structure (AT2) (cf. Figure 4c), and the second is a nearly perfect triangular lattice (TN) (cf. Figure 4f).

The general conclusions which can be extracted from our simulations are as follows: (i) the increase of metal atom size, σ_*m*_, changes its maximum coordination number due to the geometric effects. (ii) the mixture composition can “change” the maximum coordination number of the metal atom owing to the possibility of soft-interactive “gluing” with one another. This, in consequence, leads to the deterioration of the observed ordered structures.

Based on our observations, we can estimate the possible on-surface behavior of di-substituted polyphenyl-like compounds with metal atoms in different conditions. We have shown the possible paths on how molecules can assemble. We believe that those findings can be very useful for experimentalists to design future experimental conditions for a target development of particular networks of interest.
